# Identification of Active and Spectator Sn Sites in Sn-β Following Solid-State Stannation, and Consequences for Lewis Acid Catalysis

**DOI:** 10.1002/cctc.201500545

**Published:** 2015-09-01

**Authors:** Ceri Hammond, Daniele Padovan, Abbas Al-Nayili, Peter P Wells, Emma K Gibson, Nikolaos Dimitratos

**Affiliations:** [a] Cardiff Catalysis Institute, Cardiff University School of ChemistryMain Building, Park Place, Cardiff, CF10 3AT (UK) E-mail: hammondc4@cardiff.ac.uk; [b] UK Catalysis Hub, Research Complex at HarwellOxon, OX11 0FA (UK); [c] Department of Chemistry, University College LondonGordon Street, London, WC1H 0AJ (UK)

**Keywords:** biomass, heterogeneous catalysis, Lewis acids, tin, zeolites

## Abstract

Lewis acidic zeolites are rapidly emerging liquid-phase Lewis acid catalysts. Nevertheless, their inefficient synthesis procedure currently prohibits greater utilization and exploitation of these promising materials. Herein, we demonstrate that Sn^IV^-containing zeolite beta can readily be prepared both selectively and extremely rapidly by solid-state incorporation (SSI) method. Through a combination of spectroscopic (XRD, UV/Vis, X-ray absorption, magic-angle spinning NMR, and diffuse reflectance infrared Fourier transform spectroscopy) studies, we unambiguously demonstrate that site-isolated, isomorphously substituted Sn^IV^ sites dominate the Sn population up to a loading of 5 wt % Sn. These sites are identical to those found in conventionally prepared Sn-beta, and result in our SSI material exhibiting identical levels of intrinsic activity (that is, turnover frequency) despite the threefold increase in Sn loading, and the extremely rapid and benign nature of our preparation methodology. We also identify the presence of spectator sites, in the form of Sn^IV^ oligomers, at higher levels of Sn loading. The consequences of this mixed population with regards to catalysis (Meerwein–Pondorf–Verley reaction and glucose isomerization) are also identified.

## Introduction

Lewis acid catalysts have found widespread applicability for a number of liquid-phase oxidation and isomerization reactions.^1^,^2^ Amongst this class of catalysts are Lewis acidic zeolites, which are important catalysts in the area of sustainable chemistry.^3^–^5^ These crystalline, porous, inorganic Lewis acids possess several major advantages over conventional homogeneous analogues, such as AlCl_3_ and ZnCl_2_. In addition to the practical benefits of solid catalysts, which simplify downstream processing and various aspects of process intensification, the incorporation of Lewis acidic centers into hydrophobic frameworks inhibits hydrolysis and deactivation of the Lewis acid in the aqueous phase, and thereby allows these promising materials to be utilized as catalysts for a range of emerging aqueous-based reactions.^6^

Of particular interest is Sn–zeolite-β. With promising results obtained by many groups for the isomerization of glucose to fructose,^7^–^12^ the Baeyer–Villiger (BV) oxidation of ketones to lactones using H_2_O_2_ as green oxidant,^4^,^13^,^14^ Meerwein–Pondorf–Verley (MPV) transfer hydrogenations,^15^,^16^ and various other emerging catalytic transformations,^17^–^19^ the potential of Sn-β has attracted significant industrial interest. Nevertheless, some significant practical hurdles currently curtail industrial exploitation. Amongst these limitations are 1) the lengthy and complicated hydrothermal synthesis procedure, 2) the low amount of active metal incorporated per kilogram of final catalyst, resulting in low space–time yields, and 3) the large crystallite sizes obtained via typical hydrothermal synthesis, resulting in mass-transfer issues.

Given the lack of a widely applicable and scalable preparation methodology, significant academic and industrial research has recently focused on the development of new methodologies for preparing Sn-β.^19^–^24^ Recently, Hammond and co-workers^20^ demonstrated how simple, post-synthetic modifications of commercially available zeolites could readily enhance the availability and practicality of Lewis acidic zeolites for sustainable catalytic transformations.^20^ Dealumination of a commercially available β zeolite with HNO_3_ was found to lead to a highly siliceous framework possessing vacant tetrahedral (T)-sites, into which Sn^IV^ could be placed by means of solid-state incorporation (SSI) with tin(II) acetate. Not only does this approach avoid the long synthesis timescales associated with conventional Sn-β synthesis procedures (the total time for mechanochemical and heat-treatment steps is only 8 h), but it also allows for the synthesis of a material with significantly smaller crystallite sizes, increased crystallinity, greater metal content, and improved catalytic performance. Indeed, SSI Sn-β was previously found to be up to one order of magnitude more active on a space-time-yield scale than conventionally prepared Sn-β both for BV oxidations and the conversion of dihydroxyacetone into ethyl lactate.^20^ Furthermore, aqueous/liquid solutions of Sn are also avoided, and complicated/sensitive vapour deposition methods are avoided.

In our original communication,^20^ characterization of SSI Sn-β was investigated by means of UV/Vis and Raman spectroscopy, and preliminary catalytic data focusing on BV oxidation and ethyl lactate synthesis was obtained. However, for a more thorough evaluation of SSI Sn-β, and to fully compare its catalytic performance relative to the conventional analogue, further kinetic studies of additional catalytic reactions, such as the isomerization of glucose and MPV transfer hydrogenation, are required. Additionally, advanced characterization of SSI Sn-β with more sensitive spectroscopic techniques, such as X-ray absorption spectroscopy (XAS) and solid-state magic-angle spinning (MAS) NMR, is essential. Only by performing these studies can a full comparison between conventional Sn-β, and that prepared by SSI, be obtained. In this manuscript, we extend our investigations of this particular catalytic material, and demonstrate both through catalytic and spectroscopic methodologies that SSI Sn-β possesses identical site speciation and activity to conventional Sn-β at loadings up to 5 wt %, which are a factor of three higher than can be obtained by conventional hydrothermal synthesis methods. Nevertheless, some inactive sites are formed at higher loadings of Sn. The identity of these spectator sites, and their consequences for Lewis acid catalysis, is rationalized with XAS, MAS NMR, UV/Vis, XRD and diffuse reflectance infrared Fourier transform spectroscopy studies (DRIFTS).

## Results and Discussion

**Figure 1:**
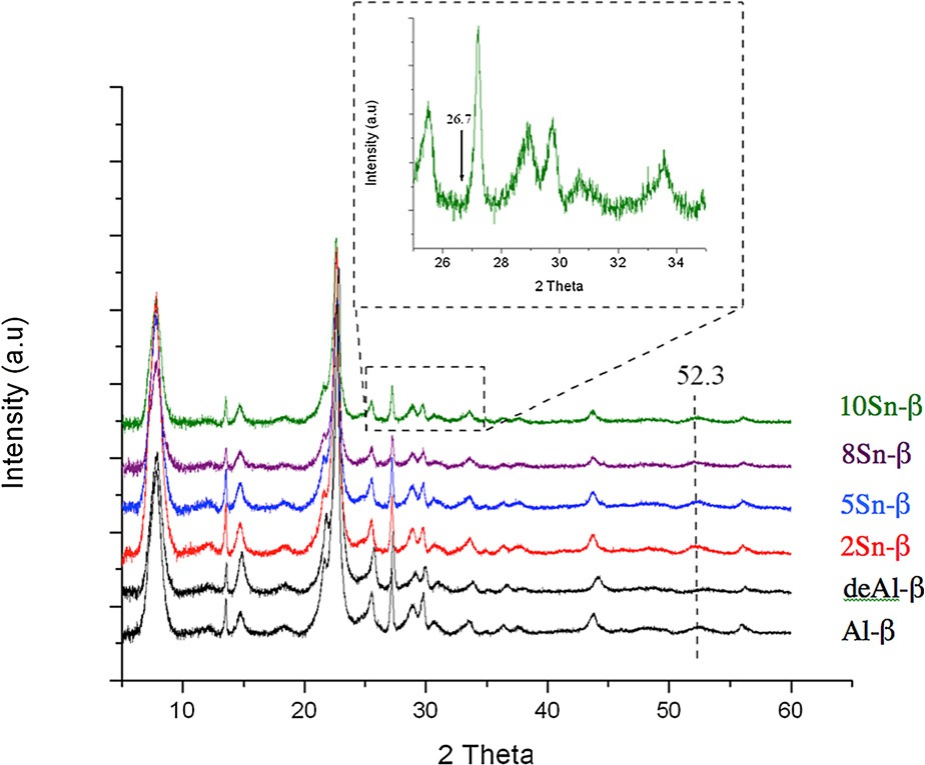
pXRD patterns of various Sn-β catalysts prepared by SSI, and containing different Sn contents. The inset gives an enlargement of the indicated spectral region of 10 Sn-β catalyst.

According to a modified version of our original synthesis protocol,^20^ we prepared a range of Sn-β catalysts with nominal loadings between 2 and 10 wt % Sn by SSI. In Table 1, the various physical and chemical properties of the synthesized catalysts and their preliminary catalytic activity for MPV transfer hydrogenation are presented. Complete dealumination of Al-β (Zeolyst, experimentally determined SiO_2_/Al_2_O_3_=26.5) was achieved by treatment in HNO_3_ following established literature protocols (13 m HNO_3_, 100 °C, 20 h, 20 mL g^−1^).^20^ In agreement to our prior studies, treatment of zeolite β with HNO_3_ does not affect the β structure, as indicated by the lack of major changes in micropore volumes (*V*_micro_, Table 1 and Table S1 in the Supporting Information), and the powder XRD (pXRD) pattern (Figure 1 and Figure S1), and results in a highly siliceous β zeolite material (SiO_2_/Al_2_O_3_>1000) possessing vacant framework sites, within which Sn^IV^ can be incorporated through post-synthetic methodologies. Sn^IV^ was subsequently introduced into these vacant sites by solid-state incorporation, by mechanochemical treatment of the dealuminated β zeolite with the appropriate quantity of tin(II) acetate for 10 min in a pestle and mortar. Calcination of the resulting homogeneous powder at 550 °C subsequently yielded the final catalytic powder. Metal loadings were determined by inductively coupled plasma mass spectrometry (ICP–MS), and are consistent—within experimental error—to the nominal loadings used during preparation. Porosymmetry measurements (Table 1, Table S1) reveal that each of the synthesized materials possesses similar micropore volumes (*V*_micro_) to conventional Sn-β, although a small but steady decrease in *V*_micro_ is observed as the metal content is increased beyond 5 wt %. Clearly, increased Sn concentrations beyond this point lead to partial pore blockage, and to decreased accessibility of the micropores of zeolite β.

**Table 1:** Physical and catalytic properties of Sn-containing zeolites prepared by SSI

Catalyst	Sn wt %^[a]^ (Si/Sn)^[b]^	*V*_micro_ [cm^3^ g^−1^]^[c]^	*X*_C*y*O_ [%]^[d]^
Al-β	–	0.23	–
deAl-β	–	0.23	0
2 Sn-β	1.8 (75)	0.23	100
5 Sn-β	4.5 (29)	0.21	100
8 Sn-β	7.3 (18)	0.19	95.3
10 Sn-β	9.2 (15)	0.18	86.3
conventional Sn-β^[e]^	±1.5	0.20	95.4

[a] Determined by ICP–MS; [b] Experimentally determined Si/Sn molar ratio; [c] Micropore volume, calculated by the t-plot method; [d] MPV reaction conditions: 100 °C, 1 h, 10 mL cyclohexanone in 2-butanol (0.2 m), CyO/Sn molar ratio of 100; [e] From Ref. 15.

Preliminary characterization of the synthesized catalysts was performed with pXRD (Figure 1) and UV/Vis spectroscopy (Figure S2). Analysis of the ligand-to-metal charge transfer bands of metal-containing zeolites with diffuse reflectance UV/Vis spectroscopy (DRUV/Vis) provides a great deal of insight of the metal site speciation.^25^ The UV/Vis spectra obtained for the synthesized samples (containing between 2 and 10 wt % Sn) all exhibit a sharp maximum at approximately 219 nm, indicative of isolated, tetrahedral Sn^IV^ species that are isomorphously substituted into the zeolite framework. Each spectrum is also significantly blue-shifted with respect to bulk SnO_2_, which exhibits a broad, characteristic absorbance at 280 nm, further indicating the presence of site-isolated Sn^IV^ sites.^16^,^20^ Although the absorbance maxima remains constant in each material, there is, however, a clear shift in absorbance edge following the incorporation of greater quantities of Sn. The absorbance edge appears to shift from approximately 250 nm/5.0 eV for 2 Sn-β, to approximately 282 nm/4.4 eV in 10 Sn-β. Although the absorbance edge falls well short of that observed for bulk SnO_2_ (approximately 350 nm/3.7 eV), this shift in band edge indicates that at least some extraframework Sn species are present in these samples, although DRUV/Vis is not sufficiently sensitive to provide a quantitative value of their presence, particularly since the extinction coefficients are unknown. However, given the relative sharpness of each peak, any extraframework Sn species are likely to be oligomeric (Sn_*x*_O_*y*_) as opposed to oxidic (SnO_2_).

As described above, the dealumination of Al-β leads to no major changes in the pXRD pattern, suggesting that the β framework remains fully intact after acidic treatment in HNO_3_. However, a shift in the calculated d-spacing following dealumination (±3.93 to 3.90 Å for the main reflection in Al-β and deAl-β, respectively (Figure S1 B)) suggests that there is a contraction in unit cell volume following the removal of Al (Figure 1). Upon incorporation of Sn^IV^ into the vacant framework sites of this material by SSI of tin(II) acetate, no obvious changes in diffraction pattern are observed, although a slight re-expansion of the unit cell volume can be observed (d-spacing of 3.93 Å for 2Sn-β), which strongly indicates that Sn has occupied the vacant framework sites. SnO_2_ produces a very clear pXRD pattern, and can readily be observed by pXRD at levels of <1 wt % (detailed description in Figure S1 A). The complete absence of any SnO_2_ reflections at 26.7 and 51.8° 2*θ* in all the catalytic materials, even those loaded with 10 wt % Sn, suggests that either SnO_2_ is not present in the materials, or that any extraframework Sn domains are so small (<5 nm) and heterogeneous that they do not possess the sufficient long-range order to produce a clear diffraction pattern. Clearly, characterization of the materials with more sensitive techniques, such as MAS NMR and XAS, is required to clarify the presence or absence of extraframework Sn species.

To evaluate the activity of the synthesized catalysts, we first focused on the MPV transfer hydrogenation of carbonyl compounds such as cyclohexanone (CyO), as this is a key reaction catalyzed by Sn-β zeolites and other Lewis acidic analogues. Preliminary kinetic analysis under conditions comparable to those of Corma et al.^15^ initially revealed (Table 1) that each of the synthesized catalysts exhibits similar catalytic activity for the MPV transfer hydrogenation of cyclohexanone. In all cases, cyclohexanol and 2-butanone were the only products detected, and carbon balances above 95 % were obtained, suggesting that any potential byproducts produced below the detectability limit of the analytical instrument are present at a relatively negligible level. With the exception of 10 Sn-β, each Sn-β catalyst prepared by SSI demonstrated comparable activity and selectivity to conventional Sn-β prepared by hydrothermal synthesis, as reported by Corma and co-workers,^15^ indicating that the Sn^IV^ site speciation in each catalyst is comparable to that of conventional Sn-β.

**Figure 2:**
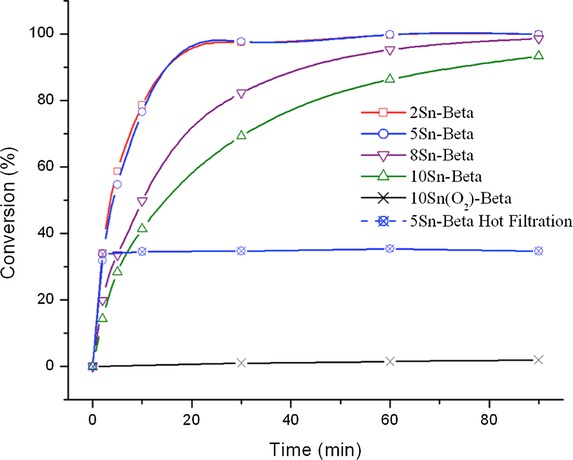
Catalytic activity of Sn-β catalysts prepared by SSI, and containing different Sn contents, for MPV hydrogenation of CyO. Reaction conditions: 100 °C, 1 h, 10 mL cyclohexanone in 2-butanol (0.2 m), CyO/Sn molar ratio of 100.

Nevertheless, more detailed kinetic analysis (Figure 2) shows this not to be the case, and demonstrates that the various catalysts possess markedly different catalytic activity. For these catalytic reactions, an identical quantity of Sn (1 mol % relative to CyO) was utilized per catalytic experiment by varying the mass of catalyst, to accurately compare specific Sn^IV^ site activity. In all cases, cyclohexanol and 2-butanone were the sole reaction products, and carbon balances were above 95 %, suggesting that Sn^IV^ loading does not impact reaction selectivity (that is, product distribution) or any adsorption effects. By examining the full kinetic profile, and particularly the early time course of the reaction, it becomes clear that that as the Sn^IV^ loading of Sn-β is increased systematically from 2 to 10 wt %, and the amount of Sn present in the reactor is kept constant, catalytic activity per mole of Sn decreases. Indeed, 8 Sn-β and 10 Sn-β are significantly less active per mole of Sn than 2 Sn-β and 5 Sn-β, which are almost identical in catalytic activity. This decrease in activity at elevated loadings strongly suggests that above Sn loadings of 5 wt %, inactive, that is, spectator, Sn^IV^ sites are produced, effectively decreasing the amount of active Sn present in the reactor, and resulting in decreased catalytic activity. To gain further critical insight into the influence of Sn^IV^ loading and catalytic activity, we also explored the catalytic activity of Sn-β for catalytic glucose isomerization, which is also catalyzed by framework Sn^IV^ sites in zeolite β, reportedly through an intramolecular 1,2-hydride shift mechanism.^8^,^26^,^27^

**Figure 3:**
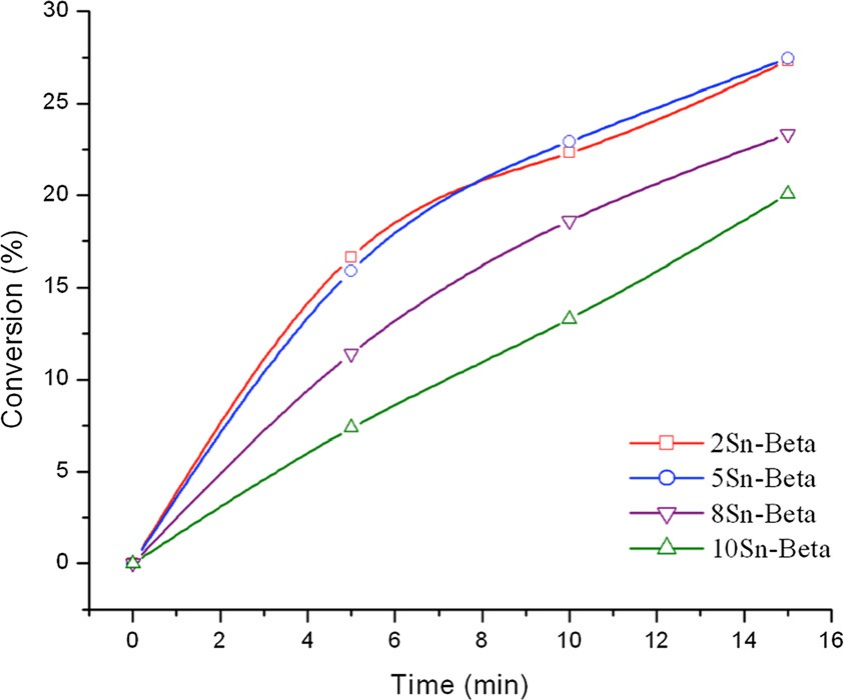
Catalytic activity of various Sn-β catalysts prepared by SSI, and containing different Sn contents, for the isomerization of glucose. Reaction conditions: 110 °C, 0.5 h, 10 wt % glucose solution (±0.61 m); glucose/Sn molar ratio of 50.

In Figure 3 the time online kinetic profile for glucose isomerization over the SSI Sn-β series is presented, whilst in Table 2 glucose conversion, fructose and mannose yields, and carbon balance for each catalytic reaction is given at levels close to iso-conversion. Reactions were performed under identical conditions to those described by Roman-Leshkov et al.^9^ Only catalytic data up to ±25 % conversion is displayed, as a significant increase in byproduct (humin) formation and a large decrease in carbon balance was observed above this level of conversion. In good agreement to the MPV reaction, both 2 Sn-β and 5 Sn-β catalyze the reaction to a similar extent, with >25 % conversion readily achieved in 15 min of reaction time under the conditions employed. The initial turnover frequencies (TOF, calculated as moles of substrate converted per mole of Sn, as determined by ICP–MS, per hour) determined in both of these cases (112 and 102 h^−1^, respectively) are almost identical, if not slightly higher, to the value calculated from the original report of Roman-Leshkov et al. (96 h^−1^).^9^ The almost identical initial TOFs observed for SSI Sn-β at loadings of up to 5 wt %, compared to open literature values, indicate that the SSI materials contain an identical active site distribution to that found in the conventional analogue. This is in good agreement with the MPV results and our initial spectroscopic data. Nevertheless, as the loading is elevated to 8 wt %, catalytic activity per mole of Sn decreases, and at a loading of 10 wt %, 10 Sn-β is considerably less reactive, strongly suggesting that spectator sites are a component of these materials at these loadings. Although it was originally proposed that framework and extraframework Sn species could be differentiated through catalytic means^10^ (by monitoring the epimerization/isomerization ratio if performing the glucose reaction in methanol), more recent studies have shown that the isomerization pathway dominates regardless of solvent choice,^11^ and that the modifications in selectivity observed in previous cases was owing to contamination of samples with alkali metal salts, such as Li^+^ and K^+^, and not differences in site speciation. Thus, no clear trends between Sn loading and selectivity were identified (Table 2) despite the differences in activities observed. In all cases, fructose was the main reaction product, and low levels of mannose formation were also observed. As such, identifying the active site ensembles present in these materials relies upon advanced spectroscopic study (see below).

**Table 2:** Glucose conversion (*X*), fructose and mannose yields (*Y*), and carbon balances (*C*) obtained during catalytic isomerization with various SSI Sn-β samples. Data is presented close to iso*-*conversion^[a]^

Catalyst	Time [min]	*X*_Gluc_ [%]	*Y*_Fru_ [%]	*Y*_Man_ [%]	*C*_bal_ [%]
2 Sn-β	10	22.3	16.4	4.4	98.6
5 Sn-β	10	22.9	15.1	3.2	95.4
8 Sn-β	10	20.0	13.1	2.9	96.0
10 Sn-β	15	20.1	13.8	3.1	96.7

[a] Reaction conditions: 110 °C, 0.5 h, 10 wt % glucose solution (±0.62 m); glucose/Sn molar ratio of 50.

**Figure 4:**
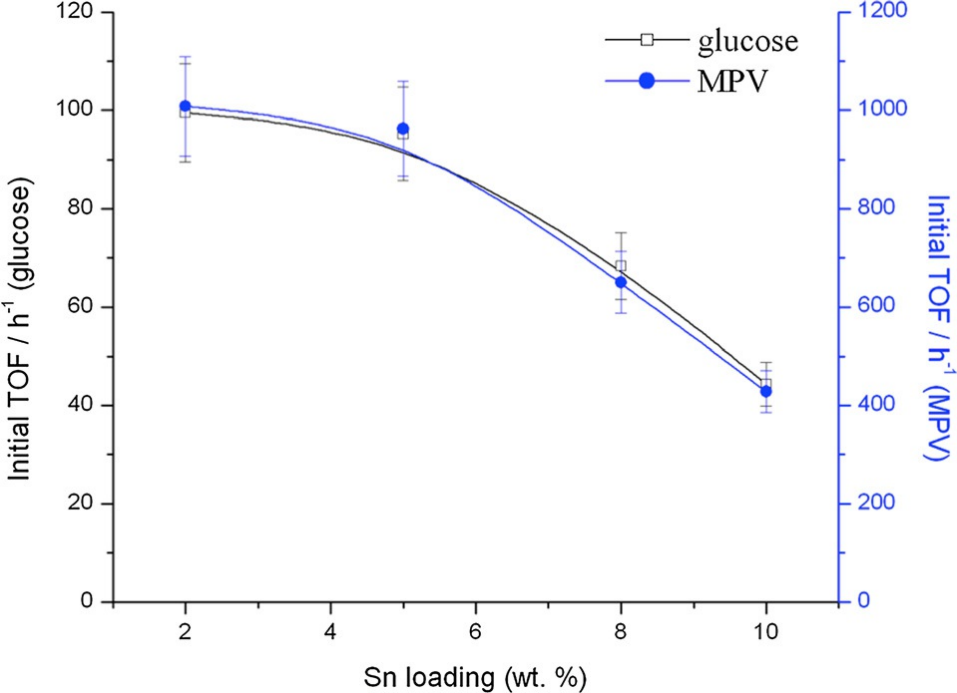
Initial TOF for each Sn-β catalyst as a function of total Sn loading for both glucose isomerization (left axis, calculated at 5 min reaction time) and MPV transfer hydrogenation (right axis, calculated at 2 min reaction time). TOF calculated as moles converted per mole Sn per hour.

To gain further insight into this decrease in activity at elevated Sn content, we calculated the initial TOF per mole of Sn in each SSI Sn-β catalyst for both catalytic reactions. Given the difficulties associated with sampling such rapid catalytic processes at such short time intervals, the experimental errors determined for these samples is considerably higher (±10 %) than those experienced during most of our catalytic protocols (typically ±5 %). Nevertheless, it is clear from Figure 4 that as Sn loading increases above ±5 wt %, there is a considerable decrease in TOF, strongly indicating that inactive Sn^IV^ sites are present in the higher loaded materials. Indeed, as the TOF of the 10 wt % sample in both cases is approximately one half of that of the 5 wt % sample, there are strong indications that potentially all of the additional Sn loading in the higher loaded materials is present in a catalytically inactive, extraframework form, or that the additional Sn loading decreases activity by other means, for example, loss of micropore access, as described above.

Noticeably, the relationship between initial activity (TOF) and Sn loading (Figure 4) is remarkably similar for both MPV transfer hydrogenation and glucose isomerization experiments. The similar TOF plots obtained strongly indicates that 1) the same active sites and species are responsible for catalytic activity in both reactions and that 2) inactive Sn^IV^ sites, possessing lower (or no) Lewis acidity, are formed in Sn-β catalysts as the loading is increased substantially beyond 5 wt %. It is thus evident that as the Sn content of SSI Sn-β is elevated above 5 wt %, spectator sites are formed during the calcination process, which negatively impact catalytic activity. We note that this is in contrast to our original communication, which had indicated that similar Sn^IV^ site activity was observed between 5 and 10 wt % samples. This deviation may be caused by several factors, but likely arises from 1) the modified synthesis procedure (see Experimental Section), which has improved the relative TOFs of the 2 and 5 wt % samples, but may change the overall Sn site speciation at higher loadings; 2) the different reactions undergoing study; and 3) the different source of zeolite precursor utilized.

Clearly the synthesis of Sn-β by SSI can lead to a material containing multiple Sn species at very high Sn loadings, although catalytic activity—and hence Sn site speciation—is remarkable similar to that found in the conventional analogue even at significantly higher, that is, a factor of three, loadings (±5 wt %). To gain further insight into the active site speciation of Sn-β as a function of loading, and to gain further insight into the nature of the active and spectator sites present, we subsequently focused upon the characterization of the Sn site speciation of SSI Sn-β with a variety of spectroscopic techniques. X-ray absorption fine structure (XAFS) analysis is a powerful technique for determining the local structure of substituted metal sites within framework architectures, with the near-edge structure (XANES) also providing information on the oxidation state. In Figure S3 the normalized Sn K edge XANES data of 2 Sn-β, 5 Sn-β, and 10 Sn-β zeolite samples are shown alongside reference spectra of SnO_2_ and Sn foil.^27^ The maxima of the first derivative of the XANES data are used to determine the position of the absorption edge. Sn foil and SnO_2_ references have edge positions of 29 200.0 eV and 29 205.1 eV, respectively. All samples have an edge position consistent with that of SnO_2_, suggesting the presence of Sn^IV^ after the 550 °C pretreatment.

The Sn K edge extended X-ray absorption fine structure (EXAFS) technique provides information about the local structure of the Sn within these samples. For completeness and clarity, we analyzed the SSI Sn-β samples both in the hydrated and dehydrated form. Whilst the majority of EXAFS analysis on metal substituted zeolites are performed following dehydration (to minimize the influence of water coordination), we feel that presenting the EXAFS data in both dehydrated and hydrated form is more suitable. The dehydrated data allows us to fully compare our results with the open literature, whilst the hydrated form is more representative of the catalytic material that operates in the liquid/aqueous phase.

**Figure 5:**
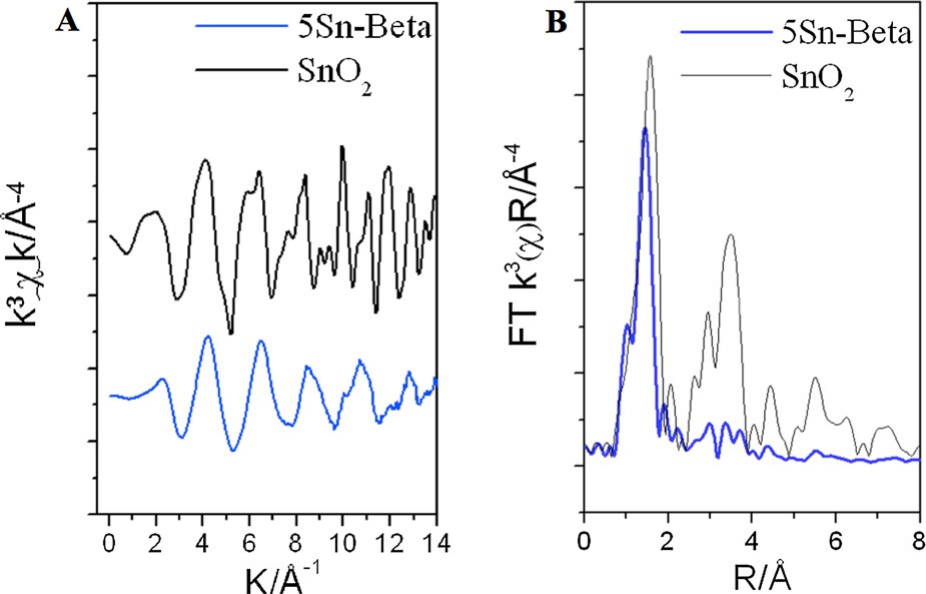
A) EXAFS *k*^3^ weighted χ data and B) magnitude of the non-phase-corrected Fourier transform, of dehydrated 5 Sn-β (blue) and SnO_2_ (black).

The EXAFS spectra of dehydrated 5 Sn-β (our optimal material in terms of loading and TOF) and the SnO_2_ reference are shown in Figure 5 A, whilst Figure 5 B presents the k^3^ weighted Fourier transform spectra of the same two samples. The spectra are of excellent quality up to and beyond a distance of 14 Å^−1^. The first features present in the FT data are assigned to Sn−O scattering interactions, whilst Sn−Sn scattering paths can be identified by the relatively intense features between 2.5 and 4 Å. SnO_2_ is also readily identified by the characteristic splitting of the oscillation around 10 Å^−1^ in the EXAFS χ data (Figure 5 A). Clearly the first peak in the FT spectrum is shifted to a lower radial distance for 5 Sn-β relative to SnO_2_. This is reflected in change in first-shell oxygen distance (Sn−O) from ±2.05 Å in SnO_2_ to 1.95 Å in 5 Sn-β, which is fully consistent with a change in geometry from octahedral to tetrahedral. This is also consistent with Sn atoms that are substituted into the vacant sites of the β framework.^28^ Between 2.5 and 4 Å there are a number of intense scattering peaks arising from Sn−Sn scattering interactions in SnO_2_. Despite their very low level, it is clear that there is a nonnegligible Sn−Sn component in 5 Sn-β following calcination at 550 °C. This can arise either from the presence of extraframework, oligonuclear Sn clusters present within the zeolite micropores (Sn_*x*_O_*y*_ species), a bulky SnO_2_ component on the external zeolite surface, or potentially through Sn–Sn pairing.^29^ Given the complete absence of SnO_2_ in all the pXRD patterns (Figure 1), the narrow full width at half maximum of the UV/Vis data (Figure S2), and the relatively low intensity of the Sn−Sn scattering pattern in the FT spectrum, it is likely that Sn–Sn interactions arise from a minor, but nonnegligible extraframework, oligomeric Sn_*x*_O_*y*_ component in 5Sn-β, although a possible contribution from Sn−Sn pairs cannot be completely excluded. The Sn−Sn coordination numbers, as derived from the EXAFS fitting, are also consistent with this hypothesis (Tables 2–3). We note that the observation of oligomeric Sn_*x*_O_*y*_ species in Sn-β is not unexpected, given that EXAFS and TEM analysis of conventional Sn-β revealed that even at low loadings (<2 wt %), conventionally prepared Sn-β contains an extraframework Sn component.^29^ Indeed, comparison of our experimental spectra to those presented by Bare et al.^29^ for conventional Sn-β indicates that an identical Sn site population is present in our material, despite the threefold increase in Sn loading. From this initial analysis, it appears that the majority of the Sn atoms in 5Sn-β are substituted into the crystalline zeolite framework, although a minor fraction of extraframework Sn_*x*_O_*y*_ is potentially present in this material. The comparable spectroscopic data obtained for our material in reference to the benchmark material is in excellent agreement with our observed catalytic data (Figures 2–4), which demonstrate that SSI Sn-β is equal to conventional Sn-β in terms of specific Sn site activity (TOF).^9^ The comparable speciation and activity of our material compared to the benchmark Sn-β material prepared by hydrothermal synthesis highlights the efficacy of our approach to preparing Sn-β, particularly since a much higher Sn loading can be achieved.

**Table 3:** EXAFS fitting data obtained for hydrated SSI Sn-β catalysts^[a]^

Catalyst	Abs Sc	*N*	*R* [Å]	2σ^2^ [Å^2^]	*E_f_* [eV]	*R*-factor
2 Sn-β	Sn−O	3.7 (3)	2.01 (1)	0.002 (2)	8 (1)	0.02
	Sn−O	1.6 (3)	2.15 (2)	0.002 (1)		
	Sn−Sn	0.7 (3)	3.24 (3)	0.005 (2)		
	Sn−Sn	0.8 (4)	3.73 (2)	0.004 (2)		
5 Sn-β	Sn−O	3.3 (4)	2.00 (1)	0.002 (1)	8 (1)	0.02
	Sn−O	2.1 (4)	2.13 (2)	0.002 (2)		
	Sn−Sn	0.6 (3)	3.24 (3)	0.005 (2)		
	Sn−Sn	0.9 (4)	3.74 (2)	0.004 (2)		
10 Sn-β	Sn−O	3.8 (8)	2.02 (1)	0.002 (2)	8 (1)	0.02
	Sn−O	1.4 (9)	2.13 (4)	0.002 (1)		
	Sn−Sn	0.9 (5)	3.23 (4)	0.006 (4)		
	Sn−Sn	1.4 (7)	3.75 (2)	0.003 (1)		

[a] Fitting parameters: 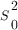
=1 as deduced by SnO_2_ standard; Fit range 2.5<*k*<13, 1<*R*<4; Number of independent points=19. Values in parenthesis give the experimental error.

**Figure 6:**
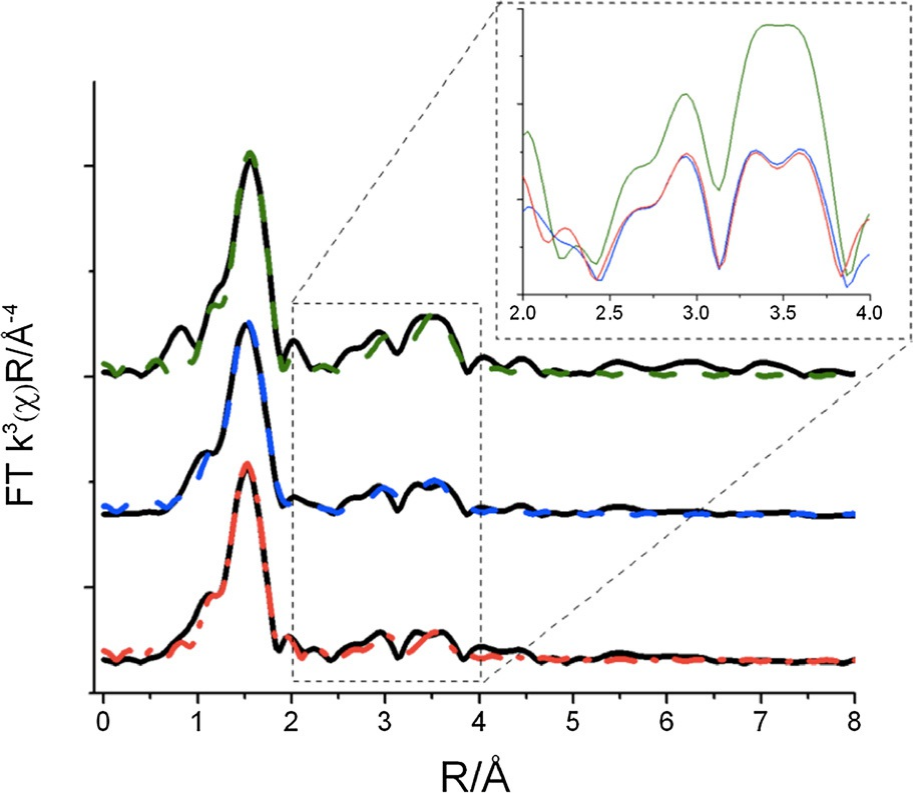
Comparison of the magnitude of the Fourier transform (FT) of hydrated Sn-β catalyst containing different loadings. From bottom to top; 2 Sn-β, 5 Sn-β, and 10 Sn-β. The fitted data is overlaid onto each spectrum; 2 Sn-β (red), 5 Sn-β (blue), and 10 Sn-β (green). The 2–4 Å region of each spectrum is overlaid and magnified in the inset.

The *k*^3^ weighted Fourier transform of hydrated 2, 5, and 10 Sn-β, along with the simulated fit, is shown in Figure 6. The χ data for these samples, with simulated fit is shown in Figure S4. *k*^3^ weighted Fourier transform of dehydrated and hydrated 5 and 10 Sn-β is in Figure S5, along with a comparison in *k*^3^ weighted FT data for dehydrated and hydrated samples. The tabulated fitting parameters for hydrated and dehydrated 2–10Sn-β are shown in Tables 3 and 4, respectively.

From Figure 6, it is evident that all the hydrated samples show the presence of Sn^IV^ substituted within the zeolitic framework alongside the copresence of Sn_*x*_O_*y*_, which is present at various levels in each material. Two Sn−O scatters are present in the data. The shortest Sn−O scattering path around 1.9–2.0 Å is assigned to Sn−O scattering interactions arising from Sn substituted into the β-zeolite framework. The longer Sn−O scattering path at a distance of 2.1 Å is associated either with Sn−O scattering interactions from Sn_*x*_O_*y*_, or water bound to the Sn within the β-zeolite framework. Upon dehydration, there is a clear decrease in intensity for the main peak, and a contraction in the first shell Sn−O distance from 2.0 to 1.95 Å (Figure S4). There is also a noticeable decrease in intensity for (or complete elimination of) the second Sn−O distance, and a decrease in the mean square disorder parameter for the oxygen shells upon dehydration. Each of these features indicates that the longer Sn−O scattering path in hydrated SSI Sn-β catalysts arises predominantly from water coordination to framework Sn^IV^ sites. However, the need for a second Sn−O scattering interaction even following dehydration (Figure 5) indicates the copresence of some Sn_*x*_O_*y*_ clusters in all the samples. This is in line with the observed Sn−Sn scattering pattern, and likely confirms that the Sn−Sn scattering path arises predominately from Sn_*x*_O_*y*_ oligomers, and less likely from Sn−Sn pairing. However, more sophisticated methods are required to completely eliminate the possibility of extensive Sn−Sn pairing in these materials.

Further information on the degree of Sn−Sn interaction for each sample can be obtained from the Sn−Sn scattering paths at ±3.2 and 3.7 Å, and the characteristic splitting of the oscillation around 10 Å^−1^ in the k^3^ weighted χ data shown in Figure S3. The inset of Figure 6 shows the overlaid FT data between 2 and 4 Å for 2, 5 and 10Sn-β. It is evident that the magnitude of the Sn−Sn scattering path is dependent on the degree of hydration, as much more intense Sn−Sn interactions are observed for the hydrated materials. Despite this, it is still possible to compare the relative intensities between all the hydrated samples. It is clear that the magnitude of Sn−Sn scattering in 2 and 5Sn-β is very similar, and this strongly suggests that both catalysts contain a comparable Sn_*x*_O_*y*_ component. This is in agreement with their observed activity, and particularly their very similar TOF values both for glucose isomerization and the MPV reaction. The comparable Sn^IV^ site speciation is also indicated by the χ data, which clearly shows that that there is only a minor splitting of the 10 A^−1^ oscillation in these two samples. This further confirms that Sn_*x*_O_*y*_ is only a minor component of these materials. We note that the extent of splitting, the magnitude of Sn−Sn interactions, and the coordination numbers for Sn−Sn interactions in both samples are almost identical (Table 4). This demonstrates that the Sn_*x*_O_*y*_ component in both catalysts is of a similar magnitude, and that the Sn_*x*_O_*y*_ component in both materials is of comparable nuclearity and composition.

**Table 4:** EXAFS fitting data obtained for dehydrated SSI Sn-β catalysts^[a]^

Catalyst	Abs Sc	*N*	*R* [Å]	2σ^2^ [Å^2^]	*E_f_* [eV]	*R*-factor
2 Sn-β	Sn−O	2.9 (2)	1.95 (1)	0.002 (1)	7 (1)	0.01
	Sn−O	1.4 (2)	2.11 (2)	0.003 (2)		
	Sn−Sn	0.7 (3)	3.24 (3)	0.007 (2)		
	Sn−Sn	0.4 (3)	3.74 (2)	0.002 (1)		
5 Sn-β	Sn−O	2.8 (2)	1.95 (1)	0.002 (1)	8 (1)	0.01
	Sn−O	1.3 (2)	2.11 (2)	0.002 (2)		
	Sn−Sn	0.4 (2)	3.25 (5)	0.008 (5)		
	Sn−Sn	0.4 (3)	3.74 (4)	0.004 (4)		
10 Sn-β	Sn−O	2.5 (2)	1.95 (1)	0.002 (2)	8 (1)	0.02
	Sn−O	2.0 (2)	2.11 (2)	0.002 (1)		
	Sn−Sn	0.7 (2)	3.25 (5)	0.005 (2)		
	Sn−Sn	0.7 (2)	3.74 (4)	0.002 (1)		
	Sn−O	2.0 (2)	2.11 (2)	0.002 (1)		

[a] Fitting parameters: 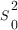
=1 as deduced by SnO_2_ standard; Fit range 2.5<*k*<13, 1<*R*<4; Number of independent points=19. Values in parenthesis are the experimental error value.

As the loading is increased beyond 5 wt %, it is clear that the Sn−Sn interactions, and hence the quantity of extraframework Sn_*x*_O_*y*_ species, increases, evidenced by increased Sn−Sn coordination numbers, an increase in the coordination number of the longer Sn−O distance, and an increase in the splitting structure shown in the χ data. This indicates that a greater amount of bulkier Sn_*x*_O_*y*_ clusters, potentially even SnO_2_, is formed at elevated loadings, accounting for at least some of the decrease in intrinsic activity observed for this sample. However, the crystallite size of this SnO_2_ must still be below ±4–5 nm, because of the absence of any SnO_2_ reflections in the pXRD pattern and the position of the absorbance edge in the UV/Vis spectra.

In the absence of full linear combination analysis, precise quantification of the framework/extraframework Sn ratio is not possible. Even so, it is evident that the increase in Sn_*x*_O_*y*_ formation at loadings above 5 wt % Sn is not substantial enough to fully account for the significant decrease in observed activity, that is, TOF. We reason that the discrepancy between the increased level of Sn_*x*_O_*y*_ formation in 10 Sn-β, and the observed TOF decrease (50 %) likely arises from a secondary effect; at a loading of 10 wt %, the micropore volume of Sn-β decreases by some 20 %, and access to the micropores, and hence the most active Sn atoms, may become somewhat restricted. This would prohibit access to some of the active Sn component, resulting in a greater-than-expected decrease in activity. Thus, not only do Sn_*x*_O_*y*_ clusters lead to a decreased number of active sites, but they also prohibit access to the existing active sites in the zeolite.

**Figure 7:**
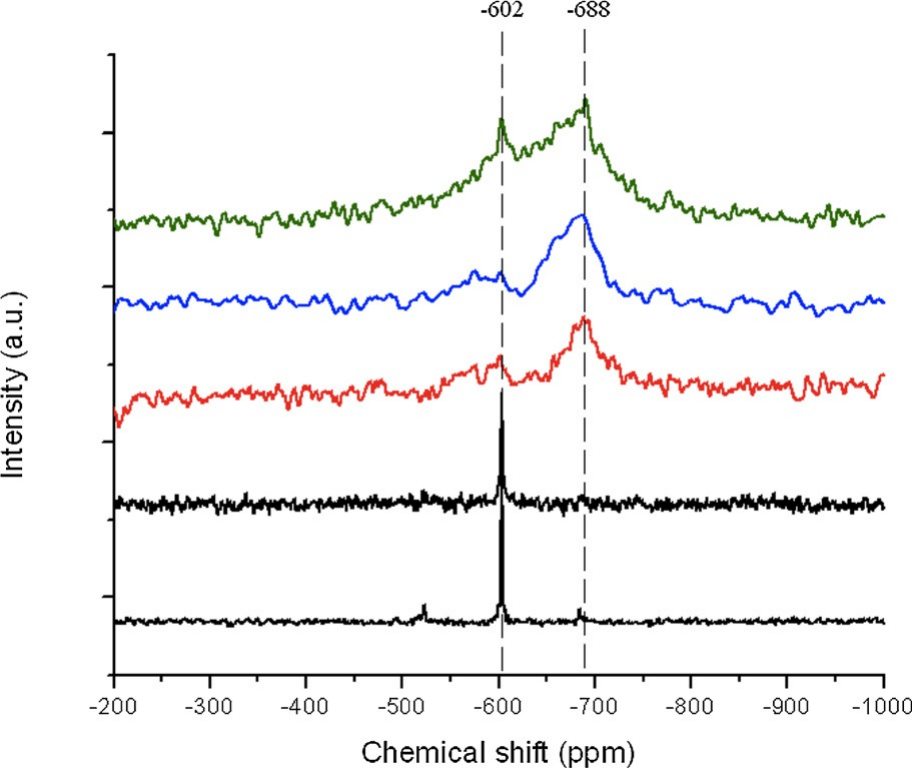
^119^Sn MAS NMR spectra of Sn-β samples containing 2 (red), 5 (blue), and 10 (green) wt % Sn. From bottom to top; bulk SnO_2_, 10 % Sn(O_2_)-β, 2 Sn-β, 5 Sn-β, and 10 Sn-β.

To further elucidate the nature of Sn species in the SSI samples, ^119^Sn MAS NMR spectroscopy was used. ^119^Sn MAS NMR spectroscopy is a sensitive technique for probing the Sn species present in Sn-containing zeolites,^10^,^11^ and by now it is well accepted that several important pieces of information can be gained from the MAS NMR spectral analysis. Hydrated, framework Sn^IV^ sites give rise to a clear resonance at *δ*=−688 ppm, with this resonance shifting to *δ*=−440 ppm upon in situ dehydration. Extraframework Sn_*x*_O_*y*_ clusters and SnO_2_ exhibit a major resonance at *δ*=−602 ppm. The magnitude of this resonance at *δ*=−602 ppm is also dependent upon the degree of Sn_*x*_O_*y*_ oligomerization, with SnO_2_ producing a MAS NMR profile with an intense resonance at *δ*=−602 ppm, and further, less intense resonances between *δ*=−500 and −700 ppm. Despite containing only natural quantities of ^119^Sn and thus exhibiting low signal-to-noise ratios, several important pieces of information can be gained from the MAS NMR spectra of the 2, 5, and 10 Sn-β samples (Figure 7). As can be seen, both 2 Sn-β and 5 Sn-β give similar MAS NMR spectra, suggesting that both materials possess a similar distribution of Sn sites. The major resonance at *δ*=−688 ppm demonstrates that hydrated, framework Sn^IV^ species predominate in these material, although the small resonances observed at *δ*=−602 ppm clearly demonstrates that some Sn_*x*_O_*y*_ domains are present in these catalysts. The absence of additional resonances between *δ*=−500 and −700 ppm indicates that the Sn_*x*_O_*y*_ domains are of low nuclearity. Each of these observations is fully consistent with, and strongly supports, our XAS analysis (see below). As the loading is further increased to 10 %, it is evident that the extraframework Sn component increases in concentration, as evidenced by a significant increase in intensity of the *δ*=−602 ppm resonance. At this loading, extraframework Sn apparently constitute a larger percentage of the Sn site speciation, although the current analysis method does not yet allow us to quantify the exact concentration of active and spectator species in this material.

**Figure 8:**
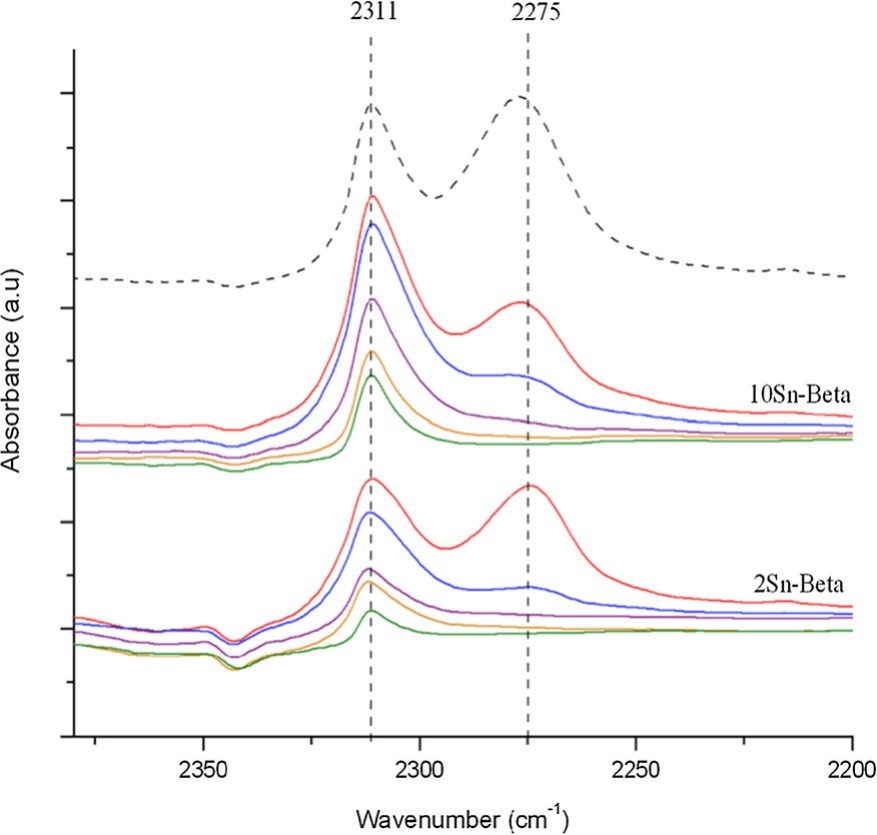
In situ CD_3_CN desorption profile for 2 Sn-β (bottom) and 10 Sn-β (middle). CD_3_CN was desorbed under a dynamic vacuum at various temperature intervals, increasing from top to bottom, and all spectra are background-referenced against the dehydrated zeolite sample. A reference spectrum of physisorbed CD_3_CN/2Sn-β is provided (dashed line).

By now it is clear that the Sn site speciation in SSI Sn-β catalysts remains relatively uniform up to a loading of 5 wt %, although some minor Sn_*x*_O_*y*_ formation is observed at these levels of Sn loading. At higher Sn loadings, increased Sn_*x*_O_*y*_ formation is observed, and larger nuclearity clusters, potentially even some SnO_2_, are also formed. To further determine whether this increased oligomer/oxide formation impacts the Lewis acidity, and hence resulting catalytic activity, of Sn-β, we subsequently investigated the Lewis acidity of the catalysts with in situ DRIFT spectroscopy. CD_3_CN is a useful molecule for probing the Lewis acid speciation in metal containing zeolites. It has been demonstrated that isolated Lewis acid sites within the zeolite framework interact with CD_3_CN, resulting in a peak in the FTIR spectrum at 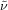
=±2310 cm^−1^, which is not observed for extraframework species.^29^–^33^ The adsorption/desorption profile for 2 Sn-β and 10 Sn-β is presented in Figure 8. Following adsorption of CD_3_CN, two major adsorption features are observed, at 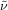
=±2270 and ±2311 cm^−1^. The first feature at 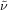
=2275 cm^−1^ is related to physisorbed CD_3_CN, weakly bound to the sample, as evidenced by its very rapid desorption upon heating. The presence of the second feature at 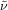
=2311 cm^−1^ in both samples arises from the CD_3_CN–Lewis acid site interaction, and demonstrates that Lewis acidic Sn^IV^ centers are present in both materials. Upon heating (up to a maximum of 200 °C), both physisorbed CD_3_CN and the CD_3_CN coordinated onto the Lewis acidic centers, gradually desorb, although the decrease in intensity for the chemisorbed species is evidently much slower. The comparable rate of desorption in both SSI samples strongly indicates that the active species in both materials possess similar levels of Lewis acid strength.

Despite this, it is evident that the normalized area of the CD_3_CN–Lewis feature, that is, the area of CD_3_CN–Lewis band divided by wt % Sn, is considerably lower for 10 Sn-β than for 2 Sn-β. Despite the semiquantitative nature of this normalization, the large difference in normalized area clearly demonstrates that the Sn_*x*_O_*y*_ species identified in 10 Sn-β by EXAFS and MAS NMR spectroscopy are unable to activate the Lewis base. Thus, they are spectator species to Lewis acid catalysis, and hence decrease the number of Lewis acid sites in the catalyst. Though our spectroscopic methods do not readily allow for quantification of the amount of spectator sites at this time, inactive sites likely account for a significant fraction of the Sn population at loadings above 5 wt %, given the significant decreases in overall TOF observed at these Sn loadings (Figure 5). This accounts for the lower activity of the higher loaded samples and further clarifies the roles of the active and spectator sites. We note that the pronounced asymmetry in the 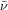
=2311 cm^−1^ band upon desorption, in particular for 2 Sn-β, may indicate that two possible bands, corresponding to two different Sn active sites with different catalytic properties, may be present in the samples. Previous FTIR studies have proposed that these may arise from “closed” (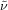
=2308 cm^−1^) and “open”/“hydrolyzed” (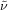
=2316 cm^−1^) Sn species that are still present in the beta framework. Given that our procedure employs dealuminated beta zeolite, which evidently contains a high density of networked silanol groups and remaining vacant framework sites, it might be expected that a preference towards „open“ Sn species may be present in this material. This may account for the apparent asymmetry of the 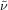
=2311 cm^−1^ band. However, further spectroscopic study is required to confirm this effect, particularly as recent work has suggested that the packing structure of CD_3_CN may inadvertently lead to the presence of multiple bands in the 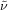
=2311 cm^−1^ region.^26^

## Conclusions

Sn-β is a material of considerable industrial interest, as it has been shown to be an important catalyst for several established and emerging sustainable chemical transformations. In this manuscript, we further demonstrate that solid-state incorporation (SSI) is a suitable alternative methodology for preparing highly active Sn-β catalysts. Not only are the resulting catalysts identical in activity to the conventional analogues, but they are prepared very rapidly, very cleanly (without, for example, solvent) and very selectivity.

In this manuscript, we have combined both catalytic and spectroscopic studies to demonstrate that isolated, framework Sn^IV^ sites dominate the Sn population of these materials, even at loadings of up to 5 wt %, which is a loading approximately two-to-three times greater than can be obtained by conventional hydrothermal methods. Identical catalytic activity to the established material is also observed, even at these significantly elevated loadings. This consequently leads to significant increases in the space–time yield of each catalyst, that is, the productivity per gram of catalyst. At higher levels of Sn loading (8–10 wt %), spectator sites in the form of Sn oligomers, and likely some SnO_2_, are also formed. Though they do not participate in the main catalytic reactions or competitive side reactions, these Sn oligomers decrease the effective concentration of active Sn in the material, and result in a catalytic powder exhibiting lower values of TOF. Spectroscopic analysis with a variety of sophisticated methods, including extended X-ray absorption fine structure, magic-angle spinning NMR, and diffuse reflectance infrared Fourier transform spectroscopy, allowed to us to obtain an overview of the active site distribution, and further allowed us to rationalize the observed catalytic trends.

## Experimental Section

### Catalyst synthesis and pretreatment

Commercial zeolite Al-β (Zeolyst, NH_4_-form, SiO_2_/Al_2_O_3_=25) was dealuminated by treatment in HNO_3_ solution (13 m HNO_3_, 100 °C, 20 h, 20 mL g^−1^ zeolite). The dealuminated powder was washed extensively with water (±500 mL g^−1^ catalyst), and dried overnight at 110 °C. Solid-state incorporation was performed by a modified procedure of Refs. <litr16 and <litr16, by grinding the appropriate amount of tin (II) acetate with the necessary amount of dealuminated zeolite for 10 min in a pestle and mortar. Following this procedure, the sample was heated in a combustion furnace (Carbolite MTF12/38/400) to 550 °C (10 °C min^−1^ ramp rate) first in a flow of N_2_ (3 h) and subsequently air (3 h) for a total of 6 h. Gas flow rates of 60 mL min^−1^ were employed at all times. The sample was held horizontally in an alumina combustion boat (10 mL capacity), and a quartz tube was used to seal the sample environment and permit gas flow.

### Catalyst characterization

Powder X-ray diffraction analysis was performed on a PANalytical X'PertPRO X-ray diffractometer, with a CuKα radiation source (40 kV and 40 mA). Diffraction patterns were recorded between 6–55° 2θ at a step size of 0.0167° (time/step=150 s, total time=1 h). DRIFT spectroscopy was performed in a Harrick praying mantis cell. The spectra were recorded on a Bruker Tensor spectrometer over a range of 4000–650 cm^−1^ at a resolution of 2 cm^−1^. In situ CD_3_CN measurements were performed on pretreated zeolite powders (550 °C, 1 h under flowing air, 60 mL min^−1^) as follows: following pretreatment, the sample was dosed with CD_3_CN vapor at room temperature for 5 min, and one spectrum was recorded. The sample chamber was subsequently evacuated under dynamic vacuum (approximately 10^−4^ mbar), and spectra were recorded at 25, 50, 100, 150, and 200 °C. All spectra were background subtracted against the pretreated zeolite. UV/Vis analysis was performed on an Agilent Cary 4000 UV/Visible spectrophotometer in diffuse reflectance mode. Samples were scanned between 190 and 900 nm at a scan rate of 600 nm min^−1^. Sn contents were determined by energy dispersive X-ray spectrometry. Specific surface area was determined from nitrogen adsorption using the BET equation, and microporous volume was determined from nitrogen adsorption isotherms using the t-plot method. Porosymmetry measurements were performed on a Quantachrome Autosorb, and samples were degassed prior to use (275 °C, 3 h). Adsorption isotherms were obtained at 77 K. MAS NMR experiments were performed at Durham University through the EPSRC UK National Solid-State NMR Service. Samples were measured under conditions identical to those reported by Bermejo-Deval and co-workers.^11^ Nonenriched Sn-β samples were measured on both a Varian VNMRS spectrometer, and a Bruker Avance III HD spectrometer with comparable performances. Both spectrometers possess operating frequencies of 400 and 149 MHz for ^1^H and ^119^Sn, respectively. Approximately 60–100 mg of sample was packed into a 4 mm rotor. Measurements were performed in direct excitation mode (spin-echo 90x-t-180y), with a recycle delay of 2 s. Samples were spun at ±12 000 Hz, and approximately 50 000 repetitions were typically employed for each sample.

### X-ray absorption spectroscopy analysis

Sn K-edge XAFS studies were performed on the B18 beamline at the Diamond Light Source, Didcot, UK. Measurements were performed using a QEXAFS setup with a fast-scanning Si (311) double crystal monochromator. The time resolution of the spectra reported herein was 2 min/spectrum (*k*_ma*x*_=14, step size 0.5 eV), on average three scans were acquired to improve the signal-to-noise level of the data for transmission measurements. All solid reference samples were diluted with cellulose and pressed into pellets to optimize the effective edge-step of the XAFS data and measured in transmission mode using ion chamber detectors. All Sn-substituted zeolite samples were prepared as undiluted pellets, with the amount of sample optimized to yield a suitable edge step and measured in transmission mode using ion chamber detectors. All XAFS spectra were acquired concurrently with the appropriate foil placed between *I_t_* and *I_ref_*. XAFS data processing was performed using IFEFFIT^34^ with the Horae package^35^ (Athena and Artemis). The amplitude reduction factor, 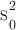, was derived from EXAFS data analysis of a known reference compound, SnO_2_, (with known coordination numbers which were fixed during analysis) to be 1.0, which was used as a fixed input parameter.

### Kinetic evaluation and analytical methods

Batch MPVO reactions were performed in a 50 mL round bottom flask equipped with a reflux condenser, which was thermostatically controlled by immersion in a silicon oil bath. The vessel was charged with a 10 mL solution of cyclohexanone in 2-butanol (0.2 m), which also contained an internal standard (biphenyl, 0.01 m), and was subsequently heated to the desired temperature (100 °C internal temperature). The reaction was initiated by addition of an appropriate amount of catalyst, corresponding to 1 mol % Sn relative to cyclohexanone. The solution was stirred at ±600 rpm with an oval magnetic stirrer bar. Aliquots of reaction solution were taken periodically for analysis, and were centrifuged prior to injection into a GC (Agilent 7820, 25 m CP-Wax 52 CB). Reactants were quantified against a biphenyl internal standard.

d-glucose (Sigma–Aldrich, ≥99 %) isomerization experiments were performed in 15 mL thick-walled glass reactors (Ace pressure tube, Sigma–Aldrich) that were heated in a temperature-controlled oil bath. The reactor was charged with 5 mL of an aqueous solution of glucose (10 wt %, 0.61 m) and an appropriate amount of catalyst corresponding to a 1:50 metal/glucose molar ratio. Once the oil had reached the desired temperature (110 °C), the reaction was initiated by vigorous stirring with a magnetic stirrer bar (600 rpm). The reactor was stirred for an appropriate length of time, and time online samples were obtained by periodically quenching the reaction by rapidly cooling the reactor in an ice bath. Aliquots of solution were extracted with a syringe, centrifuged to remove solid particulates, and were subsequently analyzed by HPLC (Agilent 1220). The compounds were separated with a Ca Hi-Plex column (6.5×300 mm, 8 m particle size, Agilent), which was isothermally held at 60 °C. Ultrapure water was used as the mobile phase, at a flow rate of 0.3 mL min^−1^. The compounds were detected by use of diode array and refractive index detectors.

## Supporting Information

As a service to our authors and readers, this journal provides supporting information supplied by the authors. Such materials are peer reviewed and may be re-organized for online delivery, but are not copy-edited or typeset. Technical support issues arising from supporting information (other than missing files) should be addressed to the authors.
