# Supplementary material for: Identification of Active and Spectator Sn Sites in Sn-β Following Solid-State Stannation, and Consequences for Lewis Acid Catalysis
Source: ChemCatChem. 2015 Sep 1;7(20):3322–31. doi: 10.1002/cctc.201500545 (PMC4641460; doi:10.1002/cctc.201500545)
Supplement: Supplementary file 1 — Supplementary [file cctc0007-3322-sd1.pdf]

Heterogeneous & Homogeneous & Bio- & Nano-
CHEMCATCHEM
CATALYSIS

Supporting Information

Identification of Active and Spectator Sn Sites in Sn-β Following Solid-State Stannation, and Consequences for Lewis Acid Catalysis

Ceri Hammond,*[a] Daniele Padovan,[a] Abbas Al-Nayili,[a] Peter. P. Wells,[b, c] Emma K. Gibson,[b, c] and Nikolaos Dimitratos[a]

cctc_201500545_sm_miscellaneous_information.pdf

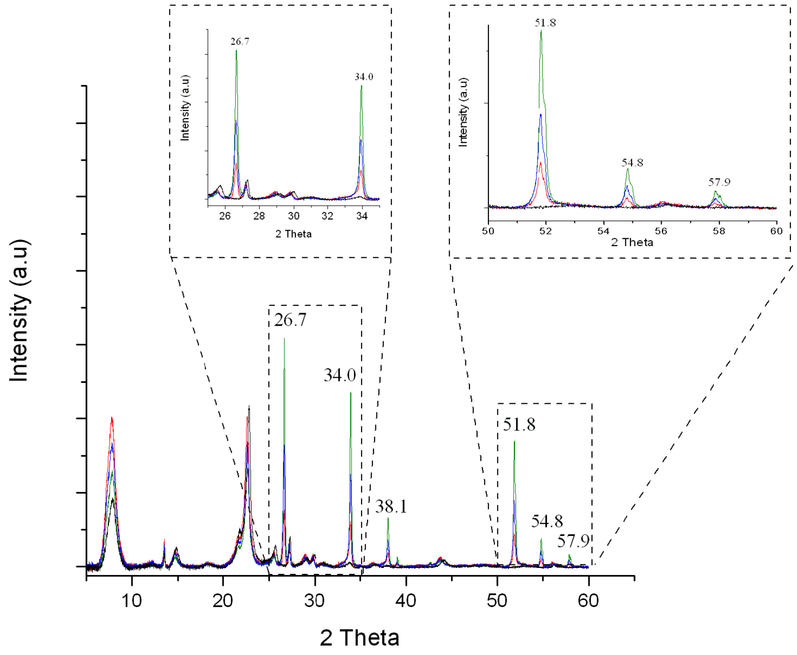

Figure S1(A). pXRD patterns of various $Sn(O_2)$-β catalysts prepared by physical mixing of SnO_2 and deAl-β, and containing different Sn contents. Black: deAl-beta; Red; $2Sn(O_2)$-β; Blue: $5Sn(O_2)$-β and Green: $10Sn(O_2)$-β. The reflections at 26.7° and 51.8° 2θ provide the best insight regarding the presence of any SnO_2, although a broad reflection at 52.3° overlaps with the 51.8° reflection to some extent. As can be seen, the SnO_2 reflections are clearly discernible at a loading of 2 % SnO_2 (red diffraction pattern).

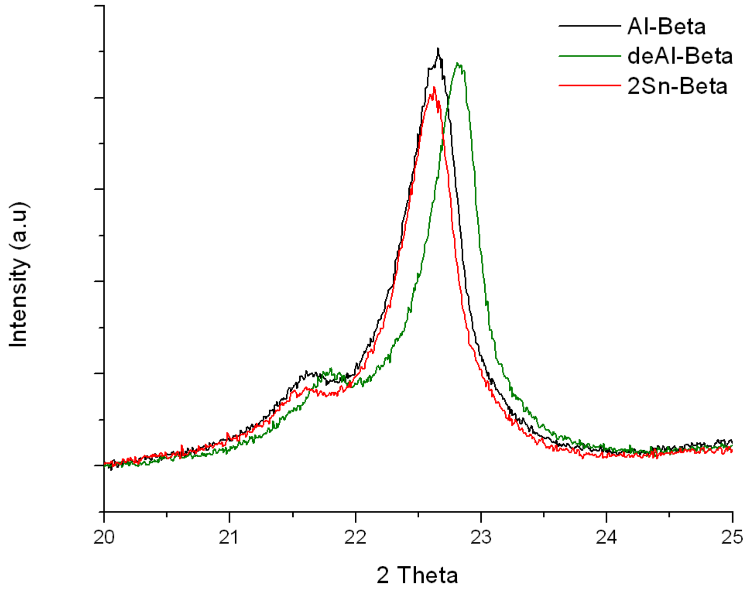

Figure S1(B). Expansion of the pXRD patterns between 20-25° 2 theta for: Black: Al-beta; Green: deAl-beta and Red; 2Sn-β. Shifts in the 2 theta position indicate a change in d-spacing and hence, unit cell volume.

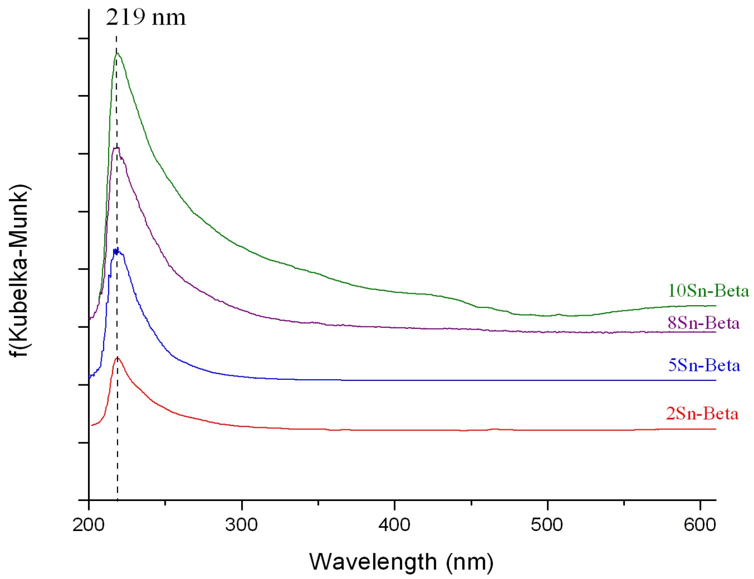

Figure S2. UV-Vis spectra of various Sn-β catalysts prepared by SSI, and containing different Sn contents. Red; 2Sn-β; Blue: 5Sn-β; Purple: 8Sn-β and Green: 10Sn-β

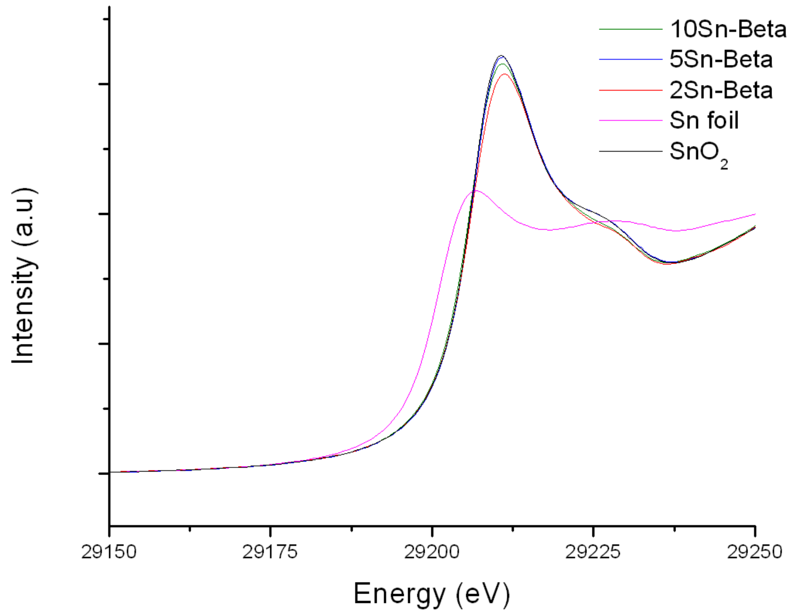

Figure S3. Normalised XANES spectra of 2Sn-β (red), 5Sn-β (blue), 10Sn-β (green). Reference XANES spectra of SnO_2 and Sn foil are included for a comparison.

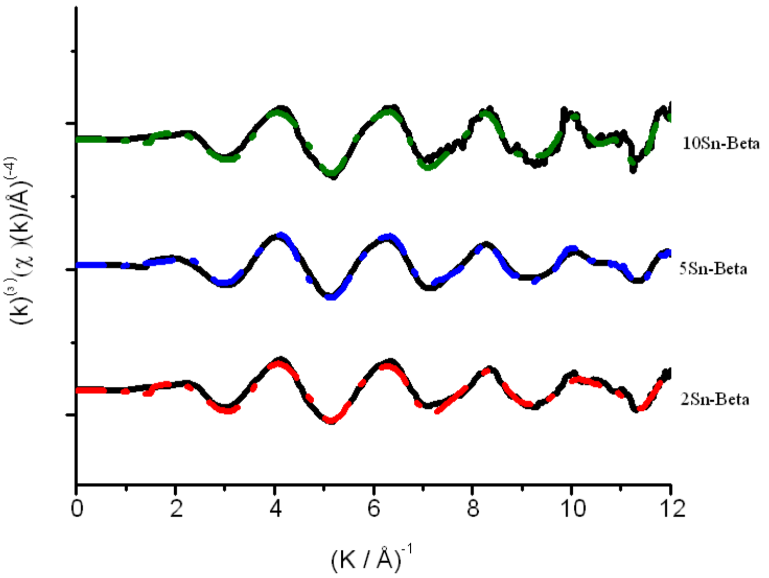

Figure S4. χ-data of hydrated Sn-β catalysts containing different Sn loadings; 2Sn-β (red), 5Sn-β (blue), 10Sn-β (green).

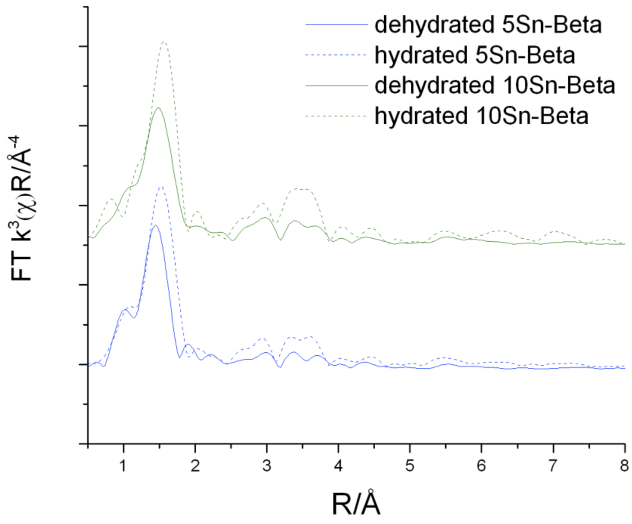

Figure S5. Comparison of the magnitude of the Fourier transform (FT) signal for dehydrated and hydrated Sn-β catalyst containing different loadings. 5Sn-β (blue), 10Sn-β (green).

Table S1. Porosymmetry data obtained for various zeolite beta materials.[a]

Sample	S_{BET} ($m^2\ g^{-1}$)[a]	$S_{external}$ ($m^2\ g^{-1}$)[b]	S_{micro} ($m^2\ g^{-1}$)[c]	V_{total} ($cm^3\ g^{-1}$)[d]	V_{micro} ($cm^3\ g^{-1}$)[e]	$V_{external}$ ($cm^3\ g^{-1}$)[f]
Al-Beta	587	91	496	0.451	0.230	0.221
de-Al-Beta	541	124	417	0.453	0.229	0.224
2% Sn-Beta	528	115	413	0.421	0.225	0.196
5% Sn-Beta	488	103	384	0.418	0.210	0.208
8% Sn-Beta	440	100	340	0.390	0.186	0.204
10% Sn-Beta	422	94	327	0.359	0.179	0.181

[a] BET surface area (S_{BET}) is calculated from the Brunauer-Emmett-Teller method; [b,c,e] the external surface area ($S_{external}$), micropore surface area (S_{micro}) and the micropore volume (V_{micro}) are calculated from the t-plot method; [d] the total pore volue (V_{total}) is evaluated at P/P_0=0.99; [f] the external pore volume ($V_{extrnal}$) is calculated according to $V_{total} – V_{micro}$.

WILEY-VCH
